# Spatial models can improve the experimental design of field‐based transplant gardens by preventing bias due to neighborhood crowding

**DOI:** 10.1002/ece3.9630

**Published:** 2022-12-14

**Authors:** Andrii Zaiats, Juan M. Requena‐Mullor, Matthew J. Germino, Jennifer S. Forbey, Bryce A. Richardson, T. Trevor Caughlin

**Affiliations:** ^1^ Boise State University Boise Idaho USA; ^2^ Carr. Sacramento Almería Spain; ^3^ U.S. Geological Survey Forest and Rangeland Ecosystem Science Center Boise Idaho USA; ^4^ USDA Forest Service, Rocky Mountain Research Station Moscow Idaho USA

**Keywords:** common garden, density dependence, gene‐by‐environment interaction, local adaptation, quantitative genetics, transplant garden

## Abstract

Field‐based transplant gardens, including common and reciprocal garden experiments, are a powerful tool for studying genetic variation and gene‐by‐environment interactions. These experiments assume that individuals within the garden represent independent replicates growing in a homogenous environment. Plant neighborhood interactions are pervasive across plant populations and could violate assumptions of transplant garden experiments. We demonstrate how spatially explicit models for plant–plant interactions can provide novel insights on genotypes' performance in field‐transplant garden designs. We used individual‐based models, based on data from a sagebrush (*Artemisia* spp.) common garden, to simulate the impact of spatial plant–plant interactions on between‐group differences in plant growth. We found that planting densities within the range of those used in many common gardens can bias experimental outcomes. Our results demonstrate that higher planting densities can lead to inflated group differences and may confound genotypes' competitive ability and genetically underpinned variation. *Synthesis.* We propose that spatially explicit models can help avoid biased results by informing the design and analysis of field‐based transplant garden experiments. Alternately, including neighborhood effects in post hoc analyses of transplant garden experiments is likely to provide novel insights into the roles of biotic factors and density dependence in genetic differentiation.

## Graphical abstract

Neighborhood interactions in common gardens: spatial models provide novel insights into spatial interactions among neighboring plants that can alter experimental outcomes. While the paper presents two approaches to the treatment of spatial interactions, it also outlines opportunities for incorporating genotype‐mediated biotic interactions into designs and analyses of common garden studies.

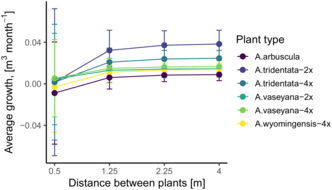

## Resumen

Los experimentos de trasplante de especies en parcelas de campo experimentales, tanto bajo condiciones ambientales comunes (“common gardens”) como diferentes (“reciprocal gardens”), son una poderosa herramienta que permite estudiar la variación genética y la interacción entre el genoma y el medio ambiente. Dichos experimentos asumen que los individuos dentro de una misma parcela representan réplicas independientes creciendo bajo condiciones ambientales homogéneas. Las interacciones entre plantas vecinas están omnipresentes en las dinámicas poblaciones y pueden suponer una violación de dichas asunciones. Sin embargo, enfoques cuantitativos que permitan evaluar la adecuación del diseño experimental son escasos. Nosotros demostramos cómo los modelos espacialmente explícitos para las interacciones planta‐planta pueden proporcionar nuevos hallazgos sobre el rendimiento genotípico en el diseño de experimentos de trasplante. Utilizamos modelos basados en individuos, junto con datos de “artemisa” (*Artemisia spp.*) procedentes de un “common garden,” para simular el impacto de las interacciones planta‐planta sobre las diferencias de crecimiento entre grupos. Encontramos que la densidad de siembra utilizada con frecuencia en muchos “common gardens” puede sesgar la estimación de la variación entre grupos. Nuestros resultados demuestran que una mayor densidad de siembra puede inflar las diferencias entre grupos, confundir la habilidad competitiva de los genomas y la variabilidad sustentada genéticamente, introduciendo así un sesgo en el experimento. Proponemos que los modelos espacialmente explícitos pueden ayudar a evitar el sesgo en los resultados mediante el apoyo en el diseño y análisis de experimentos de trasplante. Incluir efectos de vecindad en el análisis a posteriori de experimentos puede proporcionar nuevos hallazgos sobre el papel de los factores bióticos y densidad‐dependientes en la diferenciación genética.

## 1 INTRODUCTION

How environmental variation leads to genetic differences and, ultimately, to local adaptation is a fundamental question in ecology and evolution with broad implications for conservation (Aitken & Bemmels, 2016; Breed et al., 2019; Weigel & Nordborg, 2015). However, quantifying genetically underpinned differences between populations is complicated by phenotypic plasticity and environmental heterogeneity, necessitating carefully designed experiments. Transplant gardens, including common and reciprocal transplant experiments, provide a way to isolate genetic variation from phenotypic plasticity by growing individuals from different populations in the same environment (Cheplick, 2015; Johnson et al., 2022; Kawecki & Ebert, 2004; but see Galloway, 1995). By quantifying genetic variation, transplant garden experiments have resulted in transformative insights, from drivers of genetic differentiation to effective conservation policies in the face of climate change (Aitken & Bemmels, 2016; Ariza & Tielbörger, 2011).

Appropriate experimental design is key to inference on genetic variation from transplant gardens, including the assumption of independent replicates. Consequently, minimizing spatial variation that could violate experimental assumptions is paramount. Spatial variation can exist before planting due to environmental, that is, abiotic, heterogeneity. For example, variable soil fertility can alter plant fitness outcomes in planting experiments, potentially leading to biased results (Wijesinghe & Hutchings, 1997). Considerable effort has focused on minimizing the impacts of environmental heterogeneity by carefully selecting experimental sites and using block designs that account for spatial autocorrelation, for example, α‐lattice incomplete block experimental designs (Borges et al., 2019). Spatially structured variation can also emerge during the experiment due to dynamic interactions between growing plants (Urza et al., 2019). The ideal way to control for plant–plant interactions in transplant garden experiments is to choose a planting distance that ensures plants are spaced far enough to minimize any possible interactions. However, the spatial extent of plant–plant interactions is highly variable, depending on neighboring plant species and life stages (Goldberg et al., 2001; Urza et al., 2019). As a result, planting densities are rarely based on quantitative estimates of the spatial scale at which plants may interact (but see Sandquist & Ehleringer, 1997).

Inappropriately high planting densities could undermine analyses of transplant garden experiments that assume plants are independent. Decades of research from experimental and observational studies demonstrate that spatial interactions are a significant source of variation in plant performance (Adams et al., 2013; Goldberg & Fleetwood, 1987; Parachnowitsch et al., 2014). Examples of ecological mechanisms that contribute to spatial plant–plant interactions include asymmetric competition, community defense against herbivory, hydraulic redistribution, and effects of plant volatile compounds (Karban & Shiojiri, 2009; Neumann & Cardon, 2013; Ninkovic et al., 2016; Schwinning & Weiner, 1998). Pairwise interactions between neighboring plants determine the strength of spatial plant–plant interactions and depend both on distance between neighbors and their relative size (Adler et al., 2010; Barber et al., 2022). Quantifying these interactions and their consequences for plant population dynamics remains an active area of research, in part because demographic outcomes of plant–plant interactions are not always obvious (Bolker & Pacala, 1997; Law et al., 2003; Miriti, 2006). For example, weak interactions between large, established individuals may have equal or greater importance for population growth rates than strong interactions between seedlings (Caughlin et al., 2015). Despite the foundational importance of plant–plant interactions in ecology and evolution, a gap remains between quantitative models for these interactions, which are often fit using observational data, and transplant garden experiments, which often do not consider potential neighborhood effects.

This research gap is problematic as persistent effects of plant–plant interactions can lead to varying population fitness and local adaptation that transplant garden experiments aim to measure (Grassein et al., 2014; Liancourt et al., 2013; Liancourt & Tielbörger, 2009). Interactions between neighboring plants could differentially contribute to phenotypic variation of tested genotypes in transplant garden experiments (Fridley et al., 2007; Willis et al., 2010). As a result, differences in fitness that result from intraspecific interactions could be falsely attributed to genetic variation, potentially increasing Type I error in the statistical analysis; or, register as unaccounted variation in the observed data, increasing experimental error and Type II statistical error. Potential biases due to intraspecific interactions are particularly unpredictable in artificial combinations of plant genotypes that would not co‐occur in natural settings, for example, in common and transplant gardens.

Spatial models could aid inference on transplant garden experiments by providing quantitative information on the strength and scale of plant–plant interactions. Spatially explicit models for plant–plant interactions are widely used to analyze observational data on plant demography, including inter‐ versus intraspecific competition (Adler et al., 2010; Chu & Adler, 2015), the role of functional strategies in spatial interactions (Muscarella et al., 2018), and spatial clustering of genetic diversity (Shao et al., 2018). These spatially explicit models typically quantify spatial decay of competitive effects as a function of distance to neighbors, providing estimates that could directly inform the design and analysis of transplant garden experiments. Analogously, large‐scale yield experiments increasingly incorporate models for spatial autocorrelation caused by environmental heterogeneity (Burgueño, 2018). Models for spatial autocorrelation can allocate treatments before the experiment begins (“spatial designs”) or supplement analyses after the experiment ends by accounting for potential biases in results (Coelho et al., 2021; Hoefler et al., 2020; Williams & Piepho, 2013). Given the increasing interest and need to include biotic factors in genome to phenome studies (Johnson et al., 2022; Urban et al., 2016), we propose that similar spatially explicit models will be critical to isolate and account for biotic neighborhood effects.

Motivated by the theory and empirical evidence of spatial plant interactions across ecological systems (Adler et al., 2018; Caughlin et al., 2015; Law et al., 2003), our paper showcases the risks of ignoring plant–plant interactions when interpreting transplant garden experiments and provides model‐based solutions to mitigate these risks. Our spatially explicit, individual‐based modeling (IBM) approach was informed by data from a common garden study of big sagebrush (*Artemisia tridentata*), a species with high genetic diversity (Richardson et al., 2012). Using the parameters obtained from the common garden experiment, we evaluated the potential for biological interactions among individual plants to alter inference on genetic contributions to plant growth. We reproduced the spatial planting design, including distance between focal plants and their crown size, of this common garden to investigate two questions:

1. To what extent do intraspecific neighbor interactions contribute to observed population differences in plant growth?
2. How can spatial models for plant–plant interactions aid experimental design to inform appropriate planting densities?

While our case study focuses on a single species, our models are generalizable across many different species. Given the pervasive importance of plant–plant interactions for plant fitness, our approach demonstrates how spatial models could improve the design, implementation, and analysis of transplant garden experiments.

## 2 METHODS

### 2.1 Description of the common garden

To demonstrate how intraspecific density dependence affects quantitative plant traits, we parametrized the IBM based on a common garden located in central Utah, USA (Majors Flat, 39.3391, −111.5201). In this garden, big sagebrush (*Artemisia tridentata* Nutt.) source seeds were collected across the western US and outplanted in 2010 (Chaney et al., 2017). The size of the plants was monitored during the first 2 years after outplanting (Chaney et al., 2017; Richardson et al., 2021), and in our study, we used growth estimates from the second year of the experiment, calculated as the difference in crown volume between 2012 and 2011. The experimental design included 470 plants from 55 source populations randomly arranged into a grid with spacing among plants at 1 and 1.5 m on two axes. These 55 source populations represent range‐wide genetic diversity of *A. tridentata*. The experiment included three commonly recognized subspecies of *A. tridentata*, two of which differ in ploidy level, resulting in five subspecies‐cytotypes categories: *A. t. tridentata*‐2x, *A. t. tridentata*‐4x, *A. t. vaseyana*‐2x, *A. t. vaseyana*‐4x, *A. t. wyomingensis*‐4x. Akin to the language in common statistical analyses of variance, we refer to these intraspecific categories as *groups* in all subsequent analyses. Beside *A. tridentata* plants, there was also one source population of *A. arbuscula* included in the experiment.

### 2.2 Growth models

We explored the effect of intraspecific biotic interactions on plant growth in a series of spatially explicit simulations. The workflow included three steps. First, we retrieved estimated plant growth and the magnitude of spatial interactions from the models (detailed below) fit with the common garden data. Second, we used the IBM to model plant growth in simulated common gardens with varying distances between outplants. Finally, we quantified the magnitude of differences in growth among populations that could be attributed to spatial proximity among individual plants. To do that, we applied analysis of variance (ANOVA), a commonly used technique in common garden studies (Blanquart et al., 2013), using a model without the spatial term as a baseline (detailed below).

Growth model parameters were based on the subspecies‐cytotype group differences in growth rate and plant tolerance to neighborhood crowding proposed by Zaiats et al. (2021). Neighborhood crowding represents a cumulative measure of per capita effect on a quantitative trait from the immediate set of neighbors, corresponding to a measure of competition response by target plants (Chu & Adler, 2015; Goldberg & Fleetwood, 1987). The statistical growth model quantified intrinsic growth rate and crowding effect following Equations 1 and 3 (detailed below), and we refer readers to Zaiats et al. (2021) for further details on parameter estimation. Briefly, the common garden design allows for 11 distance‐to‐focal plant treatments within a 4 m neighborhood radius, while the non‐linear crowding kernel can accommodate a range of spatial neighbor effects, from a sharp to gradual declines in neighbor effect with distance. Models for plant demography in this common garden indicated different responses to neighborhood density based on subspecies identity and ploidy (i.e., cytotype) variation (Zaiats et al., 2021). Specifically, tetraploid variants demonstrated more conservative growth and greater tolerance to neighbors than their diploid variants.

### 2.3 Individual‐based modeling simulations

We ran the simulations using R software version 4.0.4 (R Core Team, 2021), and the R scripts are available from the Zenodo repository (https://doi.org/10.5281/zenodo.7411125); the *Overview, Design concepts, and Details* (ODD) description of the IBM (Railsback & Grimm, 2019) is provided in the Supporting Information (Appendix S1). Simulations of plant growth were based on the identical spatial arrangement as that in the Majors Flat common garden (i.e., the qualitative composition of plant neighborhoods), but with varying pairwise distances among individuals. We simulated 15 distance scenarios ranging from 0.5 to 4 m and propagated the uncertainty in parameter estimates (2000 posterior samples) to simulation output, resulting in 30,000 simulations. We conducted the simulations in two hypothetical scenarios: simulations that included neighbor interactions and those that did not (hereafter, *full* and *base* models; Equations 1 and 2, respectively), based on the following terms: intrinsic growth rate (i.e., the intercept, α), the effect of plant size at the beginning of the census (β), and a neighborhood crowding effect (*γ*). The crowding index (ϕ) represents a cumulative measure of neighbor crown size weighted by distance (D) from the target plant, where *c*, *b* are estimated parameters determining the strength of spatial neighbor effects. We included plant size, β Size_*i*_, as a covariate to account for initial size differences between plants and differences in growth forms among intraspecific groups (Merow et al., 2014).

(1) $${\mu^{\left(F\right)}}_{i,s}∼\text{normal}\left(\alpha_{\left[s\right]}+\beta_{\left[s\right]}{\text{Size}}_{i}+\gamma_{\left[s\right]}\phi_{i},\sigma^{2}\right)\;$$

(2) $${\mu^{\left(B\right)}}_{i,s}∼\text{normal}\left(\alpha_{\left[s\right]}+\beta_{\left[s\right]}{\text{Size}}_{i},\sigma^{2}\right)\;$$

(3) $$\phi_{i,s}=\sum_{j=1,j\neq i}^{k}\frac{c_{\left[s\right]}{\text{Size}}_{j,s}}{\exp\left(bD_{j}^{2}\right)}\;$$

where μ is the predicted growth of plant *i* belonging to group *s* with *k* neighbors, with the superscripts (*F*) and (*B*) corresponding to the full and base models, respectively, and σ is the unexplained variation. The parameters *c* and *b* in Equation 3 determine the shape of the neighborhood effect, from sharply to gradually declining with distances, which was empirically estimated to near negligible effect beyond the distance of 2.5 m away from the plant (Zaiats et al., 2021). Similar models are widely used to quantify the effect of spatial interactions on plant fitness (Adler et al., 2010; Hülsmann et al., 2020).

To check for the potential impact of a spatial pattern already existent at the time of the census (i.e., different growth and survival of individuals prior to data acquisition), we spatially perturbed the arrangement of plants and conducted an analysis of variance following the same steps as detailed above. This analysis resulted in similar estimates to those obtained using the observed starting conditions (results not shown).

### 2.4 Spatial biotic interactions

To address our first question, to what extent do intraspecific neighbor interactions contribute to group differences in plant growth, we conducted an analysis of variance using predicted growth from Equations 1 and 2 and intraspecific taxa as an independent variable. To isolate the spatial effect, we conducted this analysis on the difference between the *full* and *base* models (μ ^(*F*)^‐μ ^(*B*)^), quantifying the magnitude of change in *F*‐statistic relative to the *base* model that is explained by taxonomic groups, $\frac{F\left(\mu^{\left(\text{full}\right)}-\mu^{\left(\text{base}\right)}\right)}{F\left(\mu^{\left(\text{base}\right)}\right)}$, where *F*(.) is an *F*‐statistic of the ANOVA test performed on the response variable specified inside the parentheses. By design, this difference converges to zero when the outcomes of the *base* and *full* models are equivalent (μ ^(*F*)^ = μ ^(*B*)^) due to increasingly large distances (*D*) in the denominator of Equation 3. Additionally, we calculated Pearson's correlation coefficient (*r*) between the simulation outcomes as a function of distance among plants. Pearson's correlation represents the agreement between the two predicted scenarios, that is, including and not including neighbor interactions. For the correlation analysis, we set $\sigma^{2}$=0 in Equations 1 and 2 to propagate uncertainty only through the mean, isolating the deterministic outcomes of the simulations and varying only distance among plants. To address our second question, how can spatial models aid experimental design, we explored the spatial arrangement that would minimize the effect of biotic interactions. We calculated the scenario under which the 95% credibility interval (CI) correlation Pearson's coefficient was *r* > .99, indicating that impacts from spatial interactions among plants are unlikely to impact inference on mean group differences. The empirical estimates of plant growth using spatial and non‐spatial models are detailed in Zaiats et al. (2021) and Richardson et al. (2021).

## 3 RESULTS

The data from the common garden included 448 live plants at the time of last demographic census. We used the arrangement and size distribution from the second year after outplanting (2011) as starting conditions in the simulations. Before our demographic censuses in 2011 and 2012, 22 plants had died, and thus, gaps in the regular grid pattern existed. However, our spatial perturbation randomly rearranged the relative locations of live and dead individuals, thereby negating potential priority effects. The observed plant growth varied by the taxonomic identity of the populations, *F*_5,442_ = 44.223, *p* < .001. Specifically, populations of *A. t. tridentata* and *A. arbuscula* had the highest and lowest growth rate (i.e., change in crown volume), respectively, while populations of *A. t. vaseyana and A. t. wyomingensis* had intermediate growth.

**FIGURE 1:**
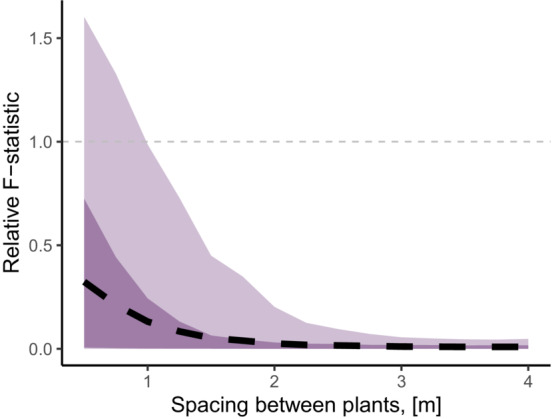
Group variation in *Artemisia tridentata* due to spatial interactions as a function of distance among plants (m) in a simulated common garden plot. The *F*‐statistic is calculated using the difference between the predicted outcomes of the full (including spatial interactions) and the base models (no spatial interactions) as an input for ANOVA. The obtained *F*‐statistic was relativized by the average *F*‐value of the base model, that is, the gray dashed line indicates an *F*‐statistic due to the spatial effects that is comparable to the group differences generated by the intrinsic growth rates. The black dashed line indicates the mean *F*‐statistic, while the darker and lighter shadows show 68% and 95% percentiles of the *F*‐statistic values in the simulated growth realizations.

As expected, the results from the simulation showed that among‐group variation in the *full* and *base* models had considerable differences (Figure 1), particularly for simulation scenarios when plants were closely arranged. The number of individual plants did not change over time, making the outcomes from the *full* and *base* models comparable. The spatial term introduced a substantial change in the simulated plant growth, and the magnitude of this change showed a strong negative relationship with distance (Figures 1 and 3). For additional details on model parameters, see Zaiats et al. (2021). The *F*‐statistic based on the difference between the *full* and *base* model outcomes (μ ^(*F*)^−μ ^(*B*)^) for the planting scenario with maximum density (pairwise distance of 0.5 m) was 32.4% greater than the *base* model (*F*_5,442_ = 128.8, 95%CI: 16.7, 254.8). The among‐group differences due to spatial interactions became negligible (i.e., converged to zero) as the spacing among plants increased.

**FIGURE 2:**
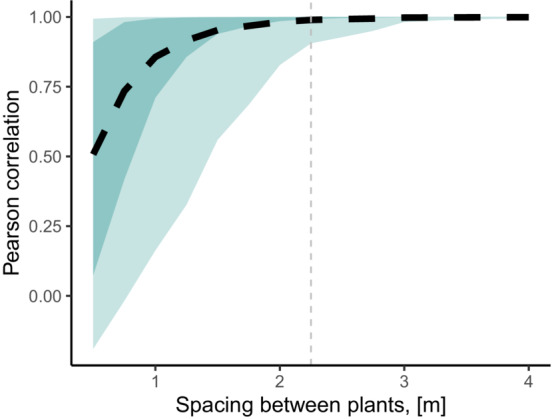
Pearson's correlation coefficient between predicted mean growth of a full (including spatial interactions) and base (no spatial interactions) models as a function of distance (m) among plants. The black dashed line indicates the average correlation coefficient, while darker‐ and lighter‐colored shades correspond to 68% and 95% error of the mean, respectively. The vertical dashed line shows the threshold where the 68% uncertainty is within Pearson's correlation > .99.

**FIGURE 3:**
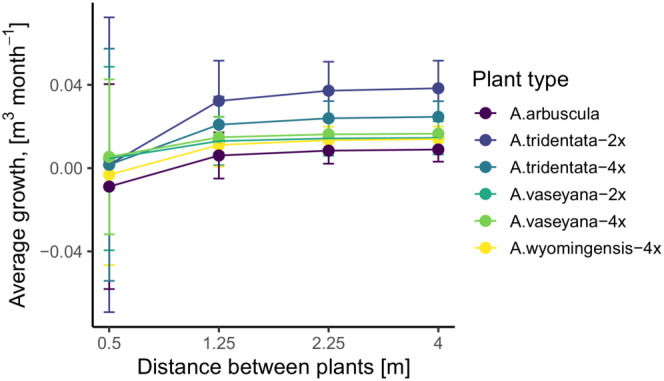
Mean differences between intraspecific groups of *Artemisia tridentata* in terms of growth. The plot shows predicted growth under two contrasting simulation scenarios: Low (0.5 m interspaces) and high (4 m interspaces) planting density. The error bars correspond to 1 standard deviation around the predicted mean.

The simulation outcomes from the *full* and *base* models showed increasing positive correlation as the distance between plants increased. The average correlation coefficient was lowest under the densest spatial arrangement (*r* = .5, 95%CI: −0.19, 0.99) but rapidly converged to *r* > .99 for distances greater than 1.25 m. For the 1 SD uncertainty around the average correlation, the *r* values varied between −.74 and .90 for the densest arrangement at 0.5 m interspaces. At this uncertainty level, the lower bound of the correlation uncertainty converged to *r* > .99 at greater than 2 m interspaces between the plants (Figure 2). When we directly compared the predicted growth for each intraspecific group under the two extreme distance scenarios, we found evidence that neighborhood interactions altered the relative performance between groups (Figures 1 and 3). These simulation results demonstrate how neighborhood interactions can change the ranking of mean performance among groups.

## 4 DISCUSSION

We have shown how spatial interactions can bias estimates of the relative performance of plant genotypes in a common garden experiment. Our simulation models reveal the dangers of assuming environmental homogeneity within transplant gardens when plants are close enough to interact with one another. In genetically diverse transplant gardens, spatial biotic interactions can create winners and losers among populations, because genotypes more vulnerable to competition should have lower performance relative to genotypes more tolerant of competition (Fridley et al., 2007). Demographic stochasticity may exacerbate these differences as some plants grow larger due to random events and become superior competitors (Hart et al., 2016). Together, our results support the need to interpret phenotypic variation in plant performance in the context of dynamic biotic conditions that evolve over time (Coleman et al., 1994).

Our work demonstrates how spatial models can identify appropriate planting distances for transplant gardens. For our focal species, big sagebrush, planting distances >1.25 m apart may be required to avoid spatial biotic interactions, even during relatively early stages of establishment (2 years after outplanting). As plants increase in size, spatial biotic interactions are likely to continue to affect the expression of demographic and functional traits of the tested genotypes (B. Richardson, *unpublished data*). A non‐exhaustive review of planting densities in highly cited transplant garden experiments reveals that spacing between plants varies markedly (*n* = 20, mean: 1.17 m, SD: 1.27 m; Table S1), including several studies with planting distances <0.1 m. While the distance required to minimize the effects of biotic interactions on plant performance will depend on a plant's size and growth rate, a quantitative approach to predict potential spatial interactions is needed. Among the 20 reviewed studies, few based planting densities on the measured spatial scale of plant–plant interactions or an explicit assumption for the relationship between plant size and biotic interactions. The motivation for minimizing plant–plant interactions via planting distances is that widely spaced plantings increase the maintenance costs of the garden, including weed suppression. However, ignoring neighborhood interactions can alter inference on the magnitude of group‐level differences.

Model‐based designs provide a way to select appropriate planting density and plant arrangement in transplant gardens. Ecologists and evolutionary biologists implementing transplant garden experiments could take inspiration from yield experiments in agronomy, where model‐based solutions to spatial autocorrelation have been thoroughly explored (Borges et al., 2019). Specifically, study designs that spatially stratify populations across potential confounding factors show superior performance compared to random plantings. In addition to abiotic spatial heterogeneity, accounting for biotic interactions using spatially explicit models provides a way to quantify neighborhood effects in existing populations across different life stages and spatial plant arrangements (Barber et al., 2022). The utility of a model‐based approach is to quantify intrinsic demographic rates and neighborhood effects, effectively separating abiotic and biotic drivers of variation in the tested genotypes (Adler et al., 2010; Zaiats et al., 2021). It is worth noting that experimental and model‐based estimates of neighborhood effects and intrinsic demographic rates may differ (Adler et al., 2018). Nevertheless, spatial models for neighborhood effects are relevant to transplant garden experiments, with utility to strengthen experimental inference while expanding the range of future research questions (Galloway, 1995; Wijesinghe & Hutchings, 1997).

An ideal way to avoid bias due to plant–plant interactions in transplant gardens would be to apply spatial models to determine appropriate planting densities before the experiment. Species‐specific data and models may be available for well‐studied species, such as big sagebrush. For other species, a lack of pre‐existing spatial data may necessitate alternate approaches to inform planting densities. Morphological traits published in the literature or measured empirically may be easier to obtain than species‐specific competition kernels (Muscarella et al., 2018; Uriarte et al., 2010). For example, Sandquist and Ehleringer (1997) used the length of lateral roots in *Encelia farinosa* to guide planting distances in a common garden. In big sagebrush, a model‐based estimation of the competition kernel in a common garden setting was also consistent with distances measured in an experiment that quantified the belowground zone of influence and potential root‐to‐root interactions (Zaiats et al., 2020, 2021). An increasing body of evidence suggests that the strength and scale of competitive interactions vary predictably with plant functional traits, including wood density and specific leaf area (Kraft et al., 2015; Uriarte et al., 2010; Yang et al., 2021). These predictive models for plant–plant interactions could guide planting densities based on trait measurements. Achieving the promise of model‐based approaches to inform transplant garden design will benefit from more dialogue between experimentalists and quantitative ecologists.

Biotic interactions in transplant garden experiments can complicate inference on genetic variation, but they also provide an opportunity to study questions that relate plant–plant interactions to genetic differentiation (Grassein et al., 2014; Johnson et al., 2022). Neighbor interactions in otherwise homogenous environments (i.e., with interspecific neighbors removed) may partially explain high variability in estimates of local adaption in studies investigating biotic and abiotic sources of genetic variation (Hargreaves et al., 2020). For example, our models revealed that competition response (i.e., neighbor tolerance) in a biotically altered transplant garden could change the relative average growth estimates (Figures 1 and 3). While plant growth alone may not be a reliable indicator of plant fitness, nor a sole basis to measure intraspecific genetic variation (Kawecki & Ebert, 2004), the majority of studies that focus on quantitative traits in transplant gardens include measurements of growth‐dependent traits that often correlate with each other (Baughman et al., 2019; Caswell, 2000; Laughlin et al., 2020; Rees & Ellner, 2016). We include only a single common garden experiment and cannot directly estimate biases in local adaptation, yet, our conclusions are relevant to replicated experiments along environmental gradients. Carefully designed treatments of biotic neighborhoods in transplant gardens could provide valuable insights into biotic factors of genetic differentiation and local adaptation.

Given the risk of biased inference from spatial plant–plant interactions, accounting for biotic neighborhood variation will likely improve future inference from transplant gardens. Existing data from transplant garden experiments (reviewed in Baughman et al., 2019; Hargreaves et al., 2020; Johnson et al., 2022) and spatially explicit models (e.g., Barber et al., 2022; Chu & Adler, 2015; Muscarella et al., 2018) provide an opportunity to determine the appropriate spacing of plants for future experiments. For example, considering the overarching importance of plant–plant interactions for population and community dynamics, growing plants in isolation from competitors is unrealistic and may give a biased view of adaptation. As an alternative to eliminating spatial interactions in transplant gardens, experimental designs could explicitly test the importance of biotic interactions by varying neighborhood composition and density as an experimental treatment, including treatments with minimal competition (Kawecki & Ebert, 2004; Münzbergová, 2007). Accounting for biotic interactions in the transplant gardens will be informative in differentiating abiotic and biotic or potential and realized ecological niches of the tested genotypes. Our study emphasizes the increasing recognition of biotic interactions as agents of population and evolutionary changes (Ariza & Tielbörger, 2011), including management interventions such as re‐introduction, translocation, or restoration efforts (Breed et al., 2019; Bucharova, 2017; McLane & Aitken, 2012; Seaborn et al., 2021). Transplant gardens with appropriate experimental designs represent an invaluable opportunity to disentangle biotic interactions and genetic diversification.

## AUTHOR CONTRIBUTIONS

**Juan Miguel Requena:** Formal analysis (equal); investigation (equal); methodology (equal); software (equal); validation (equal); visualization (equal); writing – review and editing (equal). **Matthew Germino:** Conceptualization (equal); data curation (equal); investigation (equal); methodology (equal); supervision (equal); validation (equal); writing – review and editing (equal). **Jennifer Sorensen Forbey:** Conceptualization (equal); funding acquisition (equal); investigation (equal); project administration (equal); supervision (equal); validation (equal); writing – review and editing (equal). **Bryce A. Richardson:** Conceptualization (equal); data curation (equal); funding acquisition (equal); investigation (equal); methodology (equal); validation (equal); writing – review and editing (equal). **Trevor Caughlin:** Conceptualization (equal); formal analysis (equal); funding acquisition (equal); investigation (equal); methodology (equal); project administration (equal); resources (equal); supervision (equal); validation (equal); writing – original draft (equal); writing – review and editing (equal). **Andrii Zaiats:** Conceptualization (equal); data curation (equal); formal analysis (equal); funding acquisition (equal); methodology (equal); visualization (equal); writing – original draft (equal); writing – review and editing (equal).

## CONFLICT OF INTEREST

The authors declare no conflicts of interest.

## Supporting information


Appendix S1

## Data Availability

The simulation code and data are available from the Zenodo digital repository: https://doi.org/10.5281/zenodo.7411125.
