# Supplementary material for: Spatial models can improve the experimental design of field‐based transplant gardens by preventing bias due to neighborhood crowding
Source: Ecol Evol. 2022 Dec 14;12(12):e9630. doi: 10.1002/ece3.9630 (PMC9750843; doi:10.1002/ece3.9630)
Supplement: Supplementary file 1 — Appendix S1 [file ECE3-12-e9630-s001.docx]

**Supporting Information - Appendix S1**

**Title:** Spatial models can improve the experimental design of field-based transplant gardens by preventing bias due to neighborhood crowding

**Authors:** Andrii Zaiats ([andriizaiats@u.boisestate.edu](mailto:andriizaiats@u.boisestate.edu)), Juan M. Requena-Mullor, Matthew J. Germino, Jennifer S. Forbey, Bryce A. Richardson, Trevor T. Caughlin


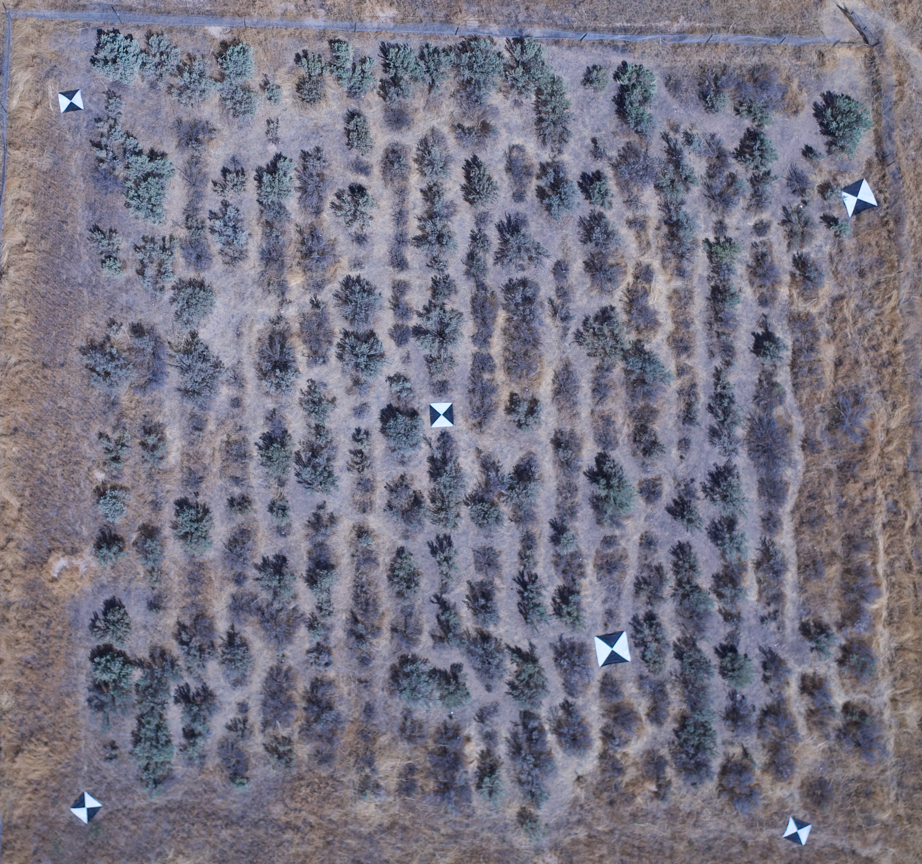


*Figure S1: Aerial image of a sagebrush common garden experiment eight years after outplanting. The photo shows a replicate of experimental design identical to the common garden used in the current study. (Photo credit: Donna M. Delparte)*

*Table S1: A subset of highly cited studies that utilized a common garden experiment. The subset is based on the Web Of Science search using the search “common garden OR reciprocal garden” published between 1900 and 2021. The table includes the first twenty studies ranked by the number of citations, and only those that included information on the spatial design of the experiment.*

| **Study** | **Life history** | **Number of species** | **Biotic treatment** | **Pairwise distance or planting density** |
| --- | --- | --- | --- | --- |
| Agrawal and Kotanen (2003) | annual, biennial, perennial grasses and forbs | 30 | removed | 1 m |
| Byers and Quinn (1998) | annual/biennial forb | 1 | intact | 81 plants/0.25 m^2^ |
| Donovan and Ehleringer (1994) | shrub | 1 | removed | 0.25 m |
| Ellison and Farnsworth (1993) | tree | 2 | removed | ≥0.1 m |
| Etterson (2004) | annual forb | 1 | removed | 12 plants/m^2^ |
| Galloway and Fenster (2000) | annual forb | 1 | intact | 0.05 m |
| Meyer and Kitchen (1994) | perennial forb | 1 | - | 0.6 x 1.2 m |
| Miller et al. (2011) | perennial forb | 5 | removed | 2 x 1 m |
| Oleksyn et al. (1998) | tree | 1 | - | 1.2 x 1.4 m |
| Rehfeldt (1993) | tree | 3 | removed | 0.3 x 0.6 m |
| Sandquist and Ehleringer (1997) | shrub | 1 | removed | 2 m |
| Schweitzer et al. (2008) | tree | 1 | - | 5 m |
| Sieman and Rogers (2001) | tree | 1 | removed | 1.5 m^2^ |
| Solbrig and Simpson (1977) | perennial forb | 1 | controlled | 4 plants/m^2^ |
| Turesson (1922) | annual forbs, perennial grasses and forbs | 17 | removed | 0.4 x 0.5 m; 0.5 x 0.6 m |
| Vergeer and Kunin (2012) | perennial forb | 1 | - | 0.1 m |
| Vitasse et al. (2009) | tree | 6 | removed | 3 x 2 m |
| Weber and Schmid (1998) | perennial forb | 2 | removed | 40 plants/m^2^ |
| Whitney et al. (2006) | annual forb | 2 | - | 0.9 m |
| Withington et al. (2006) | tree | 11 | - | 1 m |

*The ODD (Overview, Design concepts, and Details) protocol describing the IBM.*

*Purpose*

The system represented in the model is a set of plants that are stationary but change in size over time. The plants represent intraspecific diversity of *Artemisia tridentata*, with differences in growth and sensitivity to the neighbors (Chaney et al., 2017; Richardson et al., 2021; Zaiats et al., 2021). The purpose of the IBM is to explore the effect of density-dependence on plant growth and consequent group differences among intraspecific categories. This effect is measured by quantifying the change in plant size over time (*i.e.*, growth) and differences among populations in average size as a function of changing neighbor proximity.

*Entities, state variables, and scales*

The entities in the model are individual plants with the following state variables: size, location, intraspecific group (Chaney et al., 2017; Zaiats et al., 2021). The Size is recorded as the volume of above ground canopy in cubic meters [m3]. The location is an individual x and y coordinate on a relative two-dimensional grid with pairwise distances measured in meters [m]. Each plant is assigned with an intraspecific identity group. The temporal scale of the IBM process is one month, corresponding to one step in a simulation, and representing the temporal extent of the model. Therefore, the simulations match the temporal domain of the statistical models used to parametrize demographic and interaction parameters (Zaiats et al., 2021).

*Process overview and scheduling*

The process of interest is plant growth, the change in size from one timestep to the next. The process is driven by two simultaneously acting factors: (i) intrinsic growth characterized by intraspecific categories and plant size; and (ii) the cumulative measure of crowding for each plant defined by the size of neighboring plants weighted by their distance from the target plant (Equations 1 & 3 in the main text).

*Basic principles*

The model reproduces an experimental design that is frequently used in studies of quantitative genetics. In designing the IBM, we paralleled the structure and observations of the state variables that are common in the actual field experiments (Cheplick, 2015). The principle of interest is the strength of plant-plant interactions under the specifics of experimental design; we explore this process along the gradient of pairwise neighbor distances in the IBM simulations.

*Emergence*

The emergent property is the magnitude of statistical differences between intraspecific groups that can be sensitive to neighbor proximity.

*Sensing*

Each plant in the sample size experiences crowding of neighbors that intensifies with proximity and neighbor size (Chu and Adler, 2015; Zaiats et al., 2021; Figure 1).

*Interaction*

Density dependence in the current IBM does not reproduce a specific mechanism for interaction. Therefore, the presence, size, and distance to the neighbors directly affect the growth of target individuals.

*Stochasticity*

Stochasticity affects individual growth outcomes, where the magnitude of stochasticity is directly propagated from the posterior distribution of the estimated parameters in the statistical model.

*Initialization*

The initial size distribution of the plants is based on the actual plant measurements obtained in the field. For all simulation scenarios this initial size distribution was the same. The initial plant arrangement, *i.e.*, locations on the grid, also represented that of the actual experiment. Qualitatively, the collection of neighbors characterized by their intraspecific category and initial size were the same for all simulations. As part of the study, we perturbed this spatial arrangement to test for potential impact of initial plant locations on the grid, but the results were qualitatively the same as based on the original grid. The pairwise distance among individuals was according to the distance treatment in the simulations: ranging from 0.5 m to 4 m (Zaiats et al., 2021, 2020). Once the grid is assigned in the beginning of the simulation, the relative proximity among plants does not change.

*Input data*

The only input data we used for the simulations is the size of individual plants to initialize the growth.

*Submodels*

There are no submodels in the IBM.
